# Mesenchymal Stromal Cells from the Epidermis and Dermis of Psoriasis Patients: Morphology, Immunophenotype, Differentiation Patterns, and Regulation of T Cell Proliferation

**DOI:** 10.1155/2019/4541797

**Published:** 2019-12-01

**Authors:** M. E. Castro-Manrreza, L. Bonifaz, O. Castro-Escamilla, A. Monroy-García, A. Cortés-Morales, E. Hernández-Estévez, J. Hernández-Cristino, H. Mayani, J. J. Montesinos

**Affiliations:** ^1^Laboratorio de Inmunología y Células Troncales, Unidad Multidisciplinaria de Investigación Experimental Zaragoza, FES Zaragoza, Universidad Nacional Autónoma de México, Mexico City, Mexico; ^2^Unidad de Investigación Médica en Inmunoquímica, Hospital de Especialidades, Centro Médico Nacional Siglo XXI, Instituto Mexicano del Seguro Social, Mexico City, Mexico; ^3^Laboratorio de Inmunología y Cáncer, Unidad de Investigación Médica en Enfermedades Oncológicas, Centro Médico Nacional Siglo XXI, Instituto Mexicano del Seguro Social, Mexico City, Mexico; ^4^Laboratorio de Células Troncales Mesenquimales, Unidad de Investigación Médica en Enfermedades Oncológicas, Centro Médico Nacional Siglo XXI, Instituto Mexicano del Seguro Social, Mexico City, Mexico; ^5^Laboratorio de Células Troncales Hematopoyéticas, Unidad de Investigación Médica en Enfermedades Oncológicas, Centro Médico Nacional Siglo XXI, Instituto Mexicano del Seguro Social, Mexico City, Mexico

## Abstract

Psoriasis is a skin disease characterized by hyperproliferation of keratinocytes and chronic inflammation. Mesenchymal stem/stromal cells (MSCs) exhibit an immunoregulatory function that can be altered in the skin of these patients. However, to date, the presence and functional capacity of MSCs in the dermis and epidermis of patients with psoriasis have not been fully established. In the present study, we evaluated the presence of MSCs in the skin of patients by obtaining adherent cells from the dermis and epidermis of lesional and nonlesional areas and characterizing them in a comparative manner with corresponding cells obtained from the dermis (HD-MSCs) and epidermis (HE-MSCs) of healthy donors. We determined whether the adherent cells had immunophenotypic profiles and differentiation potentials that were characteristic of MSCs. In addition, we analyzed their immunosuppression function by evaluating their capacity to decrease T cell proliferation. Our results indicate the presence of MSCs in the dermis and epidermis of healthy donors and patients with psoriasis; adherent cells from all skin sources exhibited MSC characteristics, such as expression of CD73, CD90, and CD105 markers and a lack of hematopoietic and endothelial marker expression. However, the cell populations obtained showed differences in differentiation potential toward adipogenic, osteogenic, and chondrogenic lineages. In addition, we observed a low MSC obtention frequency in nonlesional epidermal samples (NLE-MSCs), which also showed alterations in morphology and proliferation rate. Interestingly, MSCs from both the nonlesional dermis (NLD-MSCs) and lesional dermis (LD-MSCs) showed higher HLA class I antigen (HLA-I) expression than HD-MSCs. Moreover, NLD-MSCs showed a low T cell proliferation suppression capacity. In summary, this study demonstrates the presence of MSCs in the epidermis and dermis of patients with psoriasis and suggests that such cells may favor the inflammatory process and thus psoriatic lesion development through high HLA-I expression and low immunosuppression capacity.

## 1. Introduction

Psoriasis is a skin disease characterized by chronic inflammation, neoangiogenesis, and keratinocyte hyperproliferation, which causes thickening of the epidermis. The pathogenesis of this disease is not yet known, but the disease is characterized by infiltration of immune system cells, such as neutrophils, macrophages, dendritic cells, and T cells, into the dermis and epidermis, as well as hyperactivation of these cells [1, 2]. In addition, proinflammatory cytokines, such as tumor necrosis factor-*α* (TNF-*α*), interferon-*γ* (IFN-*γ*), interleukin- (IL-) 2, IL-6, IL-8, IL-17A, IL-12, IL-22, and IL-23 are highly concentrated in this disease [1–3]. Therefore, inflammation has an important role in psoriasis development.

Mesenchymal stem/stromal cells (MSCs) are a population of multipotent cells that were originally identified in the bone marrow (BM) and have now been obtained from different tissues. Because there is no specific marker for these cells, certain guidelines have been established for their characterization: they must be adherent; they must express the markers CD90, CD105, CD73, CD13, and HLA-I^low^; be negative for HLA-II, CD45, CD34, CD31, and CD14; and they must have the potential for adipogenic, osteogenic, and chondrogenic differentiation [4, 5]. One of the main properties of MSCs is their ability to regulate the immune response; these cells migrate to sites of inflammation, where they are activated by proinflammatory cytokines, such as IFN-*γ*, TNF-*α*, IL-1, and IL-17 [6–8]. Once activated, MSCs begin to express and secrete various molecules that generate an anti-inflammatory environment by modulating the activation, proliferation, and differentiation of immune cells. MSCs induce differentiation of T lymphocytes and dendritic cells, which have regulatory properties [9, 10]. Additionally, they decrease NK cell activation and proliferation and affect Th1 and Th17 lymphocyte differentiation [8, 9, 11–13]. Because inflammation has an important role in the course of psoriasis, some studies have proposed the possible involvement of MSCs in the development of this disease.

Previous studies have suggested that MSCs are present in the skin of patients with psoriasis [14, 15]; however, this has not been fully established. In fact, although the inflammatory process is evident in the dermis and epidermis of patients with this disease, no study has identified and characterized the presence of MSCs in these skin layers and compared the MSCs with those obtained from healthy donors. MSC characterization is relevant because the immunoregulatory function of these cells in the skin of patients with psoriasis may be affected and therefore contribute to the pathogenesis of the disease. To the best of our knowledge, this is the first study to evaluate the presence of MSCs in the dermis and epidermis of patients with psoriasis and identify biological differences in MSC characteristics between psoriasis patients and healthy donors. The cell populations that we obtained were characterized in terms of their morphology, proliferation, immunophenotype, and differentiation potential. Furthermore, we assessed their capacity to decrease T cell proliferation to determine their immunosuppressive function. Throughout this study, we compare skin MSCs with MSCs derived from normal bone marrow (BM-MSCs), which are considered the MSC gold standard.

## 2. Methods

### 2.1. Isolation and Culture of BM-MSCs

BM samples were obtained from 5 volunteer donors following the ethical guidelines of the Villacoapa Hospital, Mexican Institute for Social Security (IMSS). Mononuclear cells were isolated from BM as previously described [16], after which the cells were resuspended in low glucose Dulbecco's modified Eagle's medium (DMEM/low glucose; Gibco BRL, Rockville, MD) that was supplemented with 10% fetal bovine serum (FBS; Gibco BRL), 4 mM L-glutamine, 100 U/mL penicillin, 100 mg/mL streptomycin, and 100 mg/mL gentamicin (all reagents were obtained from Gibco BRL); the cells were seeded at a density of 0.2 × 10^6^ cells/cm^2^ in T-25 culture flasks (Corning, Inc./Costar; New York, NY). After 4 days of culture, the nonadherent cells were removed, and fresh medium was added to the cultures. Once the cultures reached 80% confluence, the cells were harvested with trypsin (0.05% trypsin, 0.53 mM EDTA; Gibco BRL) and subcultured at a density of 0.002 × 10^6^ cells/cm^2^ in T-75 flasks (Corning, Inc./Costar). At the second passage, the cells were harvested, analyzed, and cryopreserved for future use.

### 2.2. Collection of Skin Samples and Isolation of MSCs

Skin biopsies were obtained from patients with psoriasis who came to the “Ladislao de la Pascua” dermatological center. Control skin was obtained from individuals who were admitted for gastrointestinal surgeries that were performed for reasons other than autoimmune issues at the Centro Médico Nacional Siglo XXI. In both cases, institutional ethical guidelines were followed, which included the written consent of the donors. Only one biopsy was taken from the control individuals (*n* = 5), while two samples were taken from each of the psoriasis patients (*n* = 30): one from lesional skin and one from nonlesional skin. The nonlesional skin samples were taken from a site at least 20 cm away from the lesion. Skin samples were placed overnight in a tube with RPMI 1640 culture medium (HyClone, GE Healthcare Life Science, Little Chalfont, UK) and dispase II (Protease grade II, Roche Holding AG, Basel, Switzerland). The next day, the dermis was mechanically separated from the epidermis, and both were incubated for 72 hours at 37°C and 5% CO_2_ in DMEM/low glucose supplemented with 10% fetal bovine serum, 4 mM L-glutamine, 100 U/mL penicillin, 100 mg/mL streptomycin, and 100 mg/mL gentamicin (all reagents were obtained from Gibco BRL). The culture dishes with the explants were maintained for approximately 20 days, with medium changes every 3 days. Subsequently, the adherent populations were detached with trypsin-EDTA (0.05% trypsin, 0.53 mM EDTA; Gibco BRL) and reseeded at a density of 2 × 10^3^ cells/cm^2^. The total number of cells and viability of the cultures were determined with a hemocytometer using trypan blue staining (Gibco). The cell populations obtained from the second or third passage were used for characterization of morphology, immunophenotypic profile, and differentiation capacity, and all of these characterizations were performed according to previouslydescribed protocols [16].

### 2.3. Morphologic Analysis of MSCs

To identify morphological differences between MSCs obtained from different sources, second-passage cells were grown in a Petri dish (Corning) at a density of 4000 cells/cm^2^. After 4-5 days of culture, the cells were stained with toluidine blue (Sigma-Aldrich, St. Louis, MO, USA) and examined under a phase-contrast microscope. Twenty random fields/Petri dish were scored.

### 2.4. Cell Surface Antigen Analysis of MSCs

Immunophenotypic characterization of MSCs was performed according to the methodology described by Montesinos et al. [16]. Monoclonal antibodies against surface markers characteristic of MSCs were used: CD105-PE, CD90-APC, CD73-PE, HLA-I-FITC, HLA-II-PE, and CD45-APC (BD Biosciences, San Diego, CA); CD13-PE and CD14-PE (Caltag, Buckingham, United Kingdom); and CD31-FITC and CD34-APC (Invitrogen, Carlsbad, CA). A total of 1‐1.5 × 10^6^ MSCs were resuspended in 100 mL of phosphate-buffered saline with 3% FBS and 1 mM EDTA (cytometry buffer) and incubated for 20–30 min with the appropriate antibodies. Next, the cells were washed with 1 mL of buffer and fixed with FACS Lysing Solution (BD Biosciences). The samples were analyzed on a Coulter Epics Altra Flow Cytometer (Beckman Coulter, Brea, CA), and at least 10,000 events were collected. The percentages of positive cells and mean fluorescence intensity (MFI) were obtained. The data were analyzed with FlowJo 7.6.1 software.

### 2.5. Characterization of MSC Differentiation

The differentiation capacities of the MSCs were assessed according to previously described protocols [16]. Briefly, adipogenic differentiation was induced with a Stem Cell Kit TM (Stem Cell Technologies, Inc., Vancouver, BC, Canada). The cells were incubated for 14 days in adipogenic medium, and adipogenic differentiation was determined by visualizing the presence of oil red O-stained (Sigma-Aldrich, St. Louis, MO) lipid vacuoles. Osteogenic differentiation was also induced with the Stem Cell Kit TM (Stem Cell Technologies). The cells were incubated for 21 days in osteogenic medium, and osteogenic differentiation was assessed by alkaline phosphatase staining. For chondrogenic differentiation, 2.5 × 10^5^ cells were centrifuged at 150 g for 5 min to form a pelleted cell micromass in the bottom of the tube. The precipitated cells were incubated for 28 days in chondrogenic medium (Cambrex Bio Science, Walkersville, Inc., Walkersville, MD) supplemented with 10 ng/mL transforming growth factor-*β* (Cambrex Bio Science). The resulting cell micromasses were fixed, embedded, and sliced. Cross sections were stained with Alcian blue dye (Sigma-Aldrich).

### 2.6. Coculture of MSCs and Peripheral Blood Mononuclear Cells (PBMCs)

Cocultures of MSCs and PBMCs with cell-cell contact were prepared in 24-well plates. PBMCs were obtained from peripheral blood samples from three volunteer donors via density gradient centrifugation with Ficoll-Paque Plus (specific gravity < 1.077 g/mL; GE Healthcare Bio-Sciences AB, Uppsala, Sweden). PBMCs (2 × 10^5^ cells) that had been previously stained with 5 *μ*M carboxyfluorescein succinimidyl ester (CFSE) were activated with 5 *μ*g/mL phytohemagglutinin (PHA) in the absence or presence of 1 × 10^5^ BM-MSCs, HD-MSCs, NLD-MSCs, LD-MSCs, HE-MSCs, NLE-MSCs, or LE-MSCs. After 6 days of culture, the cells were harvested to determine changes in CD3^+^ T cell proliferation via flow cytometry. The cells were examined using a FACSCanto II flow cytometer (Becton Dickinson). At least 10,000 events were collected per sample, and the data were analyzed with FlowJo 7.6.1 software.

### 2.7. Statistical Analysis

The data are expressed as the mean and standard error of the mean. Statistical analyses were performed using SPSS 20.0 software. Comparisons between groups were performed with a Mann–Whitney *U* test or paired *t*-test. A *p* value < 0.05 was considered significant.

## 3. Results

### 3.1. Presence of MSCs in the Epidermis and Dermis of the Skin from Healthy Donors and Patients with Psoriasis

**Figure 1:**
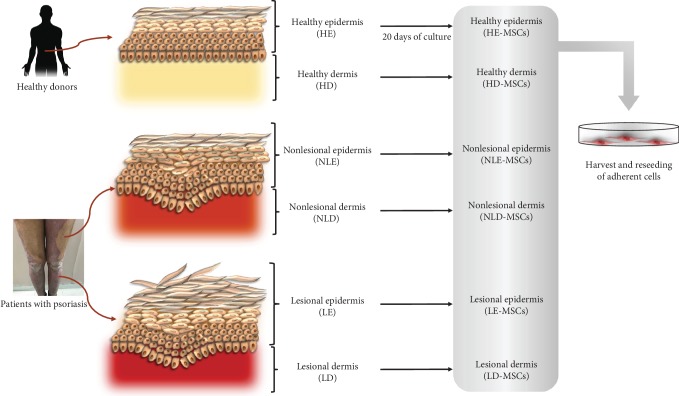
Skin sample collection. One skin sample was obtained from healthy donors (*n* = 5), while two biopsies were collected from each patient with psoriasis: one corresponding to a nonlesional area and the other to a lesional area (*n* = 30). The dermis and epidermis were isolated and processed individually to obtain adherent cells. Cells with a fibroblastoid morphology were harvested and analyzed to determine their distinctive MSC characteristics.

In the present work, we obtained adherent cells from skin samples from healthy donors and biopsies of patients with psoriasis and analyzed their morphology, proliferation, and immunophenotype as well as their capacity for adipogenic, osteogenic, and chondrogenic differentiation; all of these characteristics are present in BM-MSCs, and thus, BM-MSCs are considered the MSC gold standard and were used as a reference for comparison with other skin samples. For this purpose, skin samples were obtained from healthy donors who were undergoing abdominal surgery, and two biopsies were collected from each of the psoriasis patients: one corresponding to the nonlesional area and the other to the lesional area. The dermis and epidermis were isolated and individually processed to obtain adherent cells. Cells with a fibroblastoid morphology were harvested and analyzed to determine whether they had distinctive MSC characteristics (Figure 1).

The percentage of success in obtaining MSCs from all skin sources was variable; in the case of healthy donors, it was possible to obtain MSCs from the BM, epidermis (HE-MSCs) and dermis (HD-MSCs) in all samples processed (100%). In samples from patients with psoriasis, the MSC obtention percentages were similar for NLD-MSCs (93%), LD-MSCs (95%), and LE-MSCs (74%). However, the NLE-MSC obtention percentage was only 14%, the lowest value observed (Table 1).

### 3.2. Morphological and Proliferation Analyses of MSCs from Healthy Donors and Patients with Psoriasis

**Figure 2:**
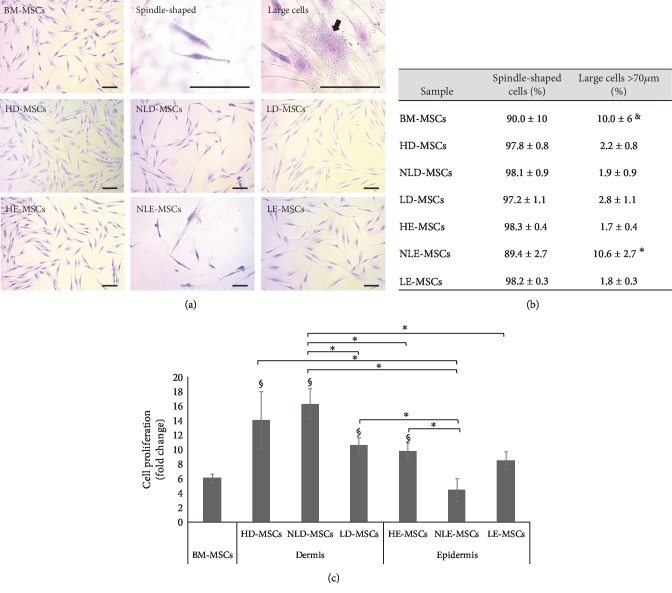
Morphology and proliferation of the MSCs obtained. (a) Representative photos of the cell morphology observed in each sample are shown (100x, scale bar = 100 *μ*m), along with representative images of cells with a spindle-shaped or large morphology (400x, scale bar = 100 *μ*m). (b) The means ± SEM of the percentage of spindle-shaped cells and large cells in the MSC cultures. ^∗^Statistically significant differences between NLE-MSCs vs. HD-, NLD-, LD-, HE-, and LE-MSCs. ^§^Statistically significant differences between BM-MSCs vs. HD-, NLD-, LD-, HE-, and LE-MSCs. (c) Cell proliferation (fold change) was measured after 100,000 cells were cultured for 6 days. After culture, the cells were harvested and counted using trypan blue staining; the viability percentage in all cultures was greater than 98%. ^∗^Statistically significant differences with *p* < 0.05. ^§^Statistically significant differences with *p* < 0.05 between BM-MSCs vs. HD-, NLD-, LD-, and HE-MSCs.

After we detected the presence of MSCs in both skin layers, we decided to analyze the biological characteristics of these MSCs that may explain their possible involvement in psoriasis development. Thus, we evaluated the morphology and proliferation rate of the MSCs. In MSC cultures, we identified small cells (length less than 70 *μ*m) with a fibroblastoid morphology (spindle-shaped cells) and large cells (length greater than 70 *μ*m) (Figure 2(a)). Interestingly, in the NLE-MSCs cultures, we detected a significant increase in the percentage of large cells (10.6% ± 2.7%; *p* < 0.05) compared with that in the HE-MSCs (1.6% ± 0.4%) and LE-MSCs cultures (1.8% ± 0.3%). Similar results were observed in HD-MSCs, NLD-MSCs, and LD-MSCs cultures (Figure 2(b)). Interestingly, large cells were observed in the BM-MSCs cultures (10.0% ± 6.0%). We also observed that the cell proliferation value (fold change) in NLE-MSCs was significantly lower (4.5 ± 1.5, *p* < 0.05) than that observed in HD-MSCs (14.0 ± 4.0), NLD-MSCs (16.3 ± 2.1), LD-MSCs (10.6 ± 1.1), and HE-MSCs (9.8 ± 1.2). In contrast, the proliferation values of the LE-MSCs (8.5 ± 1.2) and BM-MSCs (6.07 ± 0.62) were also low but were not significantly different from that of NLE-MSCs (Figure 2(c)). Notably, in one of the NLE-MSC cultures, proliferation stopped in the fourth passage.

### 3.3. Skin MSCs from Healthy Donors and Patients with Psoriasis Show Low Differentiation Capacity

**Figure 3:**
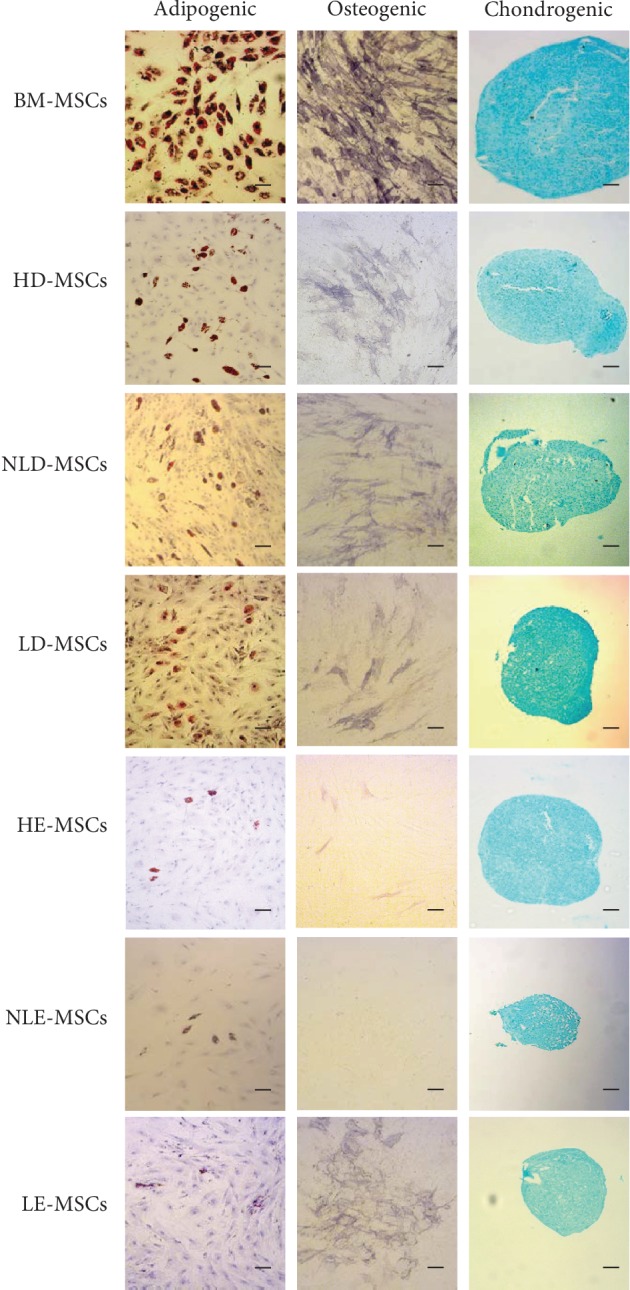
Differentiation capacity of MSCs from the skin of healthy donors and patients with psoriasis. MSCs from the different sources were cultured in adipogenic, osteogenic, or chondrogenic differentiation medium. Adipogenic differentiation was indicated by the accumulation of lipid vacuoles stained with oil red O. Osteogenic differentiation was indicated by alkaline phosphatase positivity. Chondrogenic differentiation was indicated by Alcian blue staining of glycosaminoglycans. The photos are at 100x magnification; scale bar = 100 *μ*m. A representative experiment is shown.

The differentiation capacity of MSCs from different sources was evaluated as a percentage relative to that of BM-MSCs. Thus, the following categories were assigned according to the adipogenic or osteogenic differentiation potentials that we observed: (a) high differentiation potential (50-80% of positive cells), (b) intermediate differentiation potential (30-40% of positive cells), and (c) low differentiation potential (lower than 10% of positive cells).  Figure 3 shows that the HD-MSCs, NLD-MSCs, and LD-MSCs had an intermediate potential for adipogenic and osteogenic differentiation. In contrast, the HE-MSCs and NLE-MSCs showed low adipogenic and osteogenic differentiation potentials. Interestingly, the LE-MSCs showed an intermediate osteogenic differentiation potential. Finally, chondrogenic differentiation capacity was observed in MSCs obtained from all samples (Figure 3).

### 3.4. MSCs from the Skin of Patients with Psoriasis Show Higher HLA-I Expression

**Figure 4:**
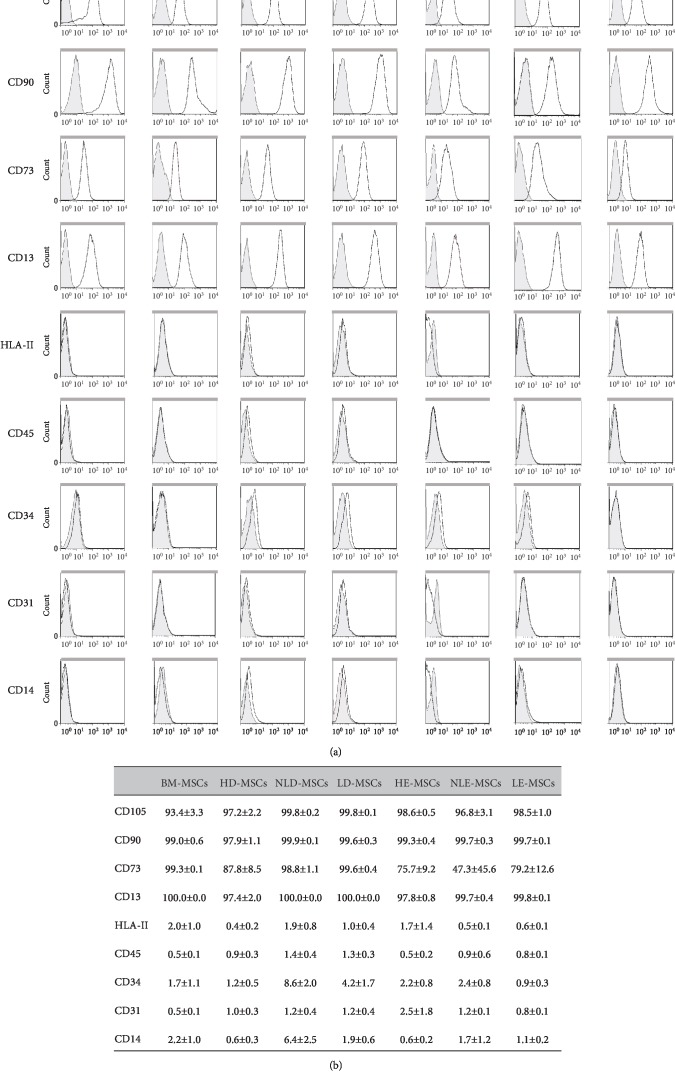
Immunophenotypic profile of MSCs from dermal and epidermal samples of healthy skin or skin with psoriasis. (a) Representative histograms of the expression of specific markers in MSCs obtained from all samples. (b) The means ± SEM of the expression percentage of each of the analyzed markers in the MSCs.

**Figure 5:**
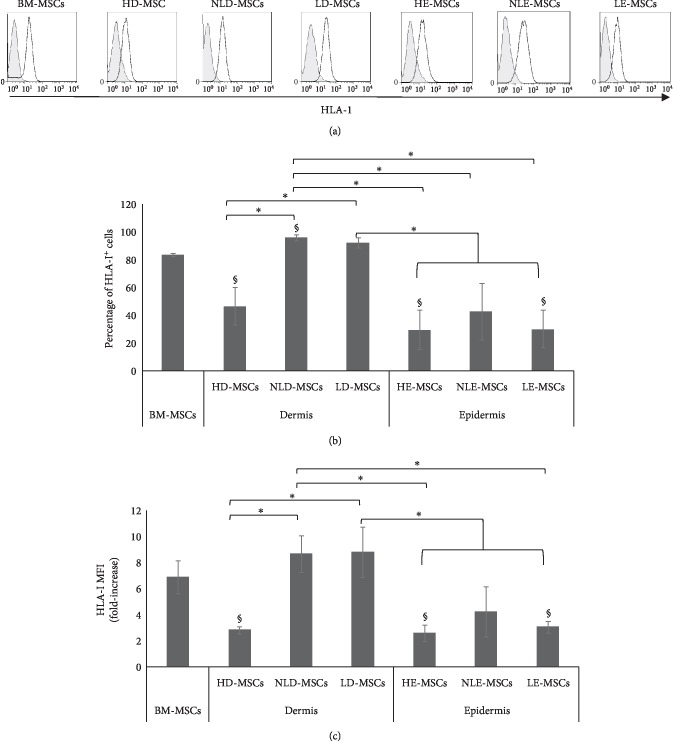
MSCs from the skin of patients with psoriasis show high HLA-I expression. (a) Representative histograms of HLA-I expression. (b) The means ± SEM of the HLA-I expression percentage. ^∗^Statistically significant differences with *p* < 0.05. ^§^Statistically significant differences between BM-MSCs vs. HD-, NLD-, HE-, and LE-MSCs; *p* < 0.05. (c) The fold increase in HLA-I expression; the means ± SEM are shown. ^∗^Statistically significant differences with *p* < 0.05. ^§^Statistically significant differences between BM-MSCs vs. HD-, HE-, and LE-MSCs, *p* < 0.05.

Cell populations were analyzed for the expression of markers that have previously been reported for BM-MSCs and for the absence of hematopoietic and endothelial markers. As shown in Figures 4(a) and 4(b), the expression levels of CD13, CD90, CD73, and CD105 in the MSCs derived from healthy donors and patients with psoriasis were similar. All the populations lacked CD45, CD34, CD31, and CD14 expression. Interestingly, we found differences in HLA-I expression; specifically, a significant increase in the percentage of HLA-I^+^ cells was observed (Figure 5(b)) in NLD-MSCs (96.0% ± 2.0%) and LD-MSCs (92.2% ± 3.5%) compared with that in HD-MSCs (46.6% ± 13.6%).

Similar changes were observed in the MFI of HLA-I (Figure 5(c)) in the NLD-MSCs (8.7-fold increase) and LD-MSCs (2.8-fold increase) compared with that in the HD-MSCs (8.8-fold increase). By contrast, the HE-MSCs, NLE-MSCs, and LE-MSCs showed low HLA-I expression (29.7% ± 14.1%, 42.9% ± 20.5%, and 30.1% ± 13.6%, respectively), and there were no significant differences between these cell populations (Figure 5).

### 3.5. MSCs from the Skin of Patients with Psoriasis Showed Low Immunoregulatory Capacity

**Figure 6:**
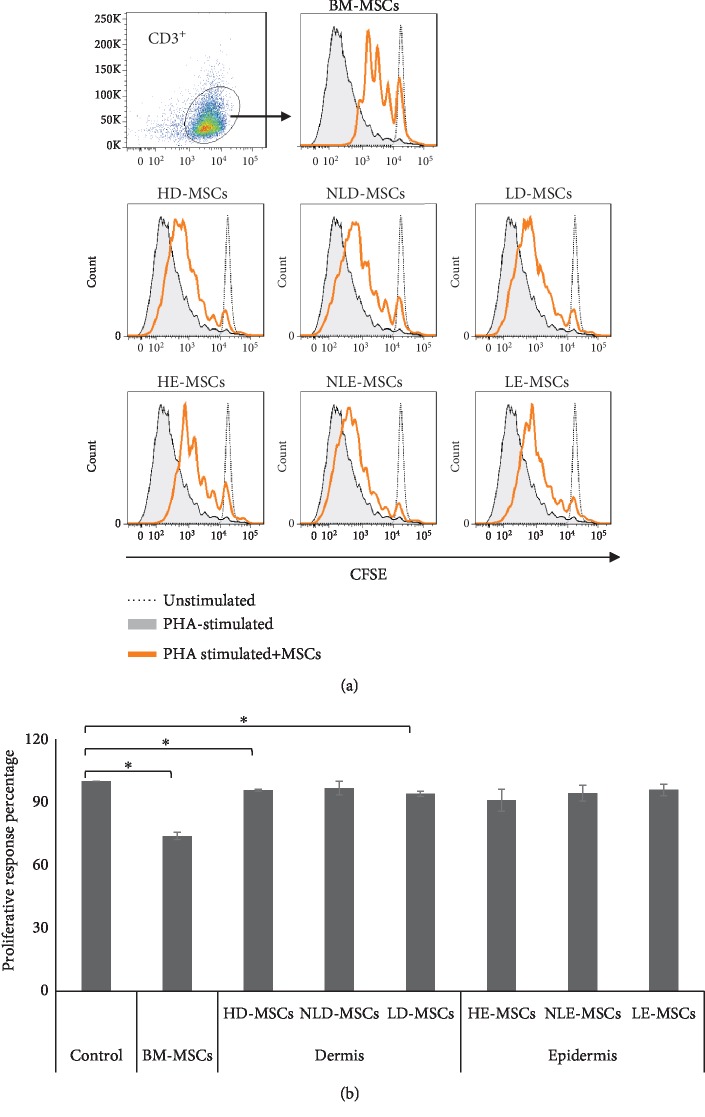
MSCs from the skin of patients with psoriasis show low immunoregulatory capacity. Cocultures were performed with 2 × 10^5^ PBMCs that were stained with CFSE and activated with PHA in the absence (proliferation positive control) or presence of MSCs that were derived from the dermis or epidermis of healthy or psoriatic skin (lesional and nonlesional areas). BM-MSCs were used as a positive control for immunoregulation. After 6 days of culture with cell-cell contact, CD3^+^ T cell proliferation was analyzed. (a) Representative histograms are shown. (b) Proliferative response percentage; the means ± SEM of 6 independent cocultures are shown. ^∗^Statistically significant differences with *p* < 0.05.

T cell infiltration and overactivation in the skin with psoriasis is a key element in the pathology of the disease. Therefore, we analyzed the ability of MSCs to decrease T cell proliferation.  Figure 6 shows that only HD-MSCs (95.6% ± 1.0%) and LD-MSCs (94% ± 1.3%) decreased CD3^+^ T cell proliferation (100% positive proliferation control), although their effect was weaker than that observed with BM-MSCs (73.9% ± 1.7%). MSCs that were obtained from other sources did not have this capacity: NLD-MSCs (96.7% ± 3.3%), HE-MSCs (90.8% ± 5.27%), NLE-MSCs (94.2% ± 3.8%), and LE-MSCs (95.7% ± 2.8%).

## 4. Discussion

Previous studies have suggested the presence of MSCs in the skin of patients with psoriasis; however, although the dermis and epidermis are known to be involved in the pathology of this disease [2], no study has analyzed the presence of these cells in both skin layers or the biological differences between MSCs derived from lesional versus nonlesional areas. Therefore, in the present work, we obtained MSCs from the dermis and epidermis of skin biopsies from healthy donors and patients with psoriasis (lesional and nonlesional areas). We performed a complete characterization of these samples by analyzing their morphology, proliferation, marker expression, trilineage differentiation capacity, and immunoregulatory function.

In our cultures, we obtained populations of adherent cells with a fibroblastic morphology that was similar to that observed in BM-MSCs. We found MSC populations in practically all the dermis and epidermis samples from healthy donors and in dermis from patients with psoriasis. However, the percentage of NLE-MSCs was low (14%).

Interestingly, adherent cells were only obtained from three nonlesional epidermis samples. A number of studies have correlated MSC morphology with the MSC capacity for proliferation [17, 18], differentiation [19, 20], immunosuppression [21], and cellular senescence [22]. Therefore, we decided to determine possible differences in the morphology of MSC populations obtained from the different sources and observed that, in addition to the low frequency of MSCs in the nonlesional epidermis, the cells that developed in these cultures showed a significant increase in the percentage of large cells and a low proliferation capacity. In the samples with the greatest potential to expand in culture, spindle-shaped cells predominated [17–22]. It is possible that in the nonlesional epidermis samples, a microenvironment that affects certain MSC characteristics is already present and that this may contribute to the development of lesions.

We observed that MSCs from the skin showed an immunophenotype that was similar to that of BM-MSCs because there were no statistically significant differences in the expression levels of the markers that were analyzed, except in the expression of HLA-I, whose relevance will be discussed later. Previous studies have described the presence of MSCs in the dermis derived from healthy donors and patients with psoriasis, although in some cases, a complete characterization has not been performed [23]. For example, a previous study reported the presence of multipotential mesenchymal stem cells in the foreskin but did not determine the expression of CD73, HLA-I, or HLA-II or the chondrogenic differentiation capacity of the cells [24]. Likewise, other studies have not analyzed differentiation potential [25, 26]. Similarly, the presence of MSCs has been reported in nonlesional, perilesional, and lesional areas of skin from patients with psoriasis, but the trilineage differentiation capacity was not determined [27, 28]. In contrast, another study suggested the presence of MSCs after observing the expression of CD105, CD73, CD90, HLA-DR, and CD45 and cell adipogenic, osteogenic, and chondrogenic differentiation capacities. The authors concluded that skin-derived MSCs have characteristics similar to those of BM-MSCs [29]. In our study, we performed a complete characterization of MSCs obtained from skin samples and found significant differences in their differentiation capacity in comparison with MSCs from the BM.

The analysis of the differentiation capacity of HD-MSCs, NLD-MSCs, and LD-MSCs showed intermediate adipogenic and osteogenic differentiation potential. This was most evident in the HE-MSCs and NLE-MSCs, in which these differentiation potentials were low. These observations are supported by previous studies of mammalian skin that report a population of multipotent adult stem cells that can be obtained only from the dermis; these cells express characteristic MSC markers but have a lower adipogenic and chondrogenic differentiation capacity than observed in MSCs derived from adipose tissue [30]. Similar results have been obtained in populations of mesenchymal progenitor cells from the skin, whose differentiation potential was lower than that of BM-MSCs [31]. Interestingly, LE-MSCs that were oil red O positive and alkaline phosphatase positive were more frequently observed in the lesional epidermis than in the nonlesional epidermis. This finding may indicate that the presence of inflammatory stimuli stimulates migration or activation of MSCs in the lesional epidermis. MSCs have been shown to secrete trophic factors that favor tissue remodeling through activation of resident stem cells in tissues [30, 32]. In this sense, it is likely that MSCs contribute to the incomplete expansion and differentiation of epidermal stem cells in lesional epidermis, which would result in epidermis thickening [14]. In this regard, it has been demonstrated that MSCs favor keratinocyte proliferation [33].

Finally, we observed that the HD-MSCs, NLD-MSCs, and LD-MSCs did not present differences in differentiation potential. These results are supported by previous studies in which MSCs from the dermis of healthy donors and patients with psoriasis were obtained; the authors reported no differences in the MSC populations that were analyzed [15, 34, 35]. Importantly, to the best of our knowledge, this is the first study that analyzes the chondrogenic differentiation capacity of MSCs obtained from skin with psoriasis (NLD-MSCs, LD-MSCs, NLE-MSCs, and LE-MSCs), and we observed chondrogenic differentiation in all the cell populations that we examined.

The expression of HLA-I in the NLD-MSCs and LD-MSCs was increased in comparison with that in HD-MSCs. Because this outcome was observed even in NLD-MSCs, it is possible that this alteration may contribute to the development of lesions in patients with psoriasis. This hypothesis is supported by previous studies that report high concentrations of vascular endothelial growth factor (VEGF), even in nonlesional skin, but this concentration was reduced after 12 weeks of treatment with TNF-*α* inhibitors [27, 36]. These results suggest that in the dermis of patients with psoriasis, even in areas where there are no obvious lesions, resident MSCs are already in an activated state due to the presence of proinflammatory cytokines, such as TNF-*α*.

The association between HLA-I and the pathogenesis of psoriasis has been well established. However, in spite of this close association, few studies have analyzed the patterns of HLA-I expression in the lesions themselves or in the different cell types that are present in the skin. To date, general mRNA analyses, particularly those of HLA-C mRNA, do not indicate differences between healthy skin and psoriatic skin [37]. Moreover, Carlen et al. analyzed the HLA-C expression levels in total protein extract and found higher HLA-C expression in lesional skin than in healthy or nonregional skin. The highest HLA expression in the skin of patients with psoriasis was primarily observed in infiltrated immune cells and in the basement membrane [38]. This study supports our results, which show an increase in HLA-I expression in NLD-MSCs and LD-MSCs, and emphasizes the importance of analyzing HLA-I expression in specific and isolated cell populations.

In psoriasis, the primary role of HLA-I is to present antigens to CD8^+^ T cells [39]. One of the proposed steps in the development of this disease involves recognition of HLA-I antigens by CD8^+^ T cells, not only in antigen-presenting cells but also in keratinocytes and other stromal cells. In this sense, the high HLA-I expression found in the NLD-MSCs that we observed in this study is important because this event may contribute to a greater recognition of antigens by CD8^+^ T cells and thus initiate and exacerbate inflammation in lesional skin. In addition, our results show that NLD-MSCs have a decreased ability to affect T lymphocyte proliferation compared with their healthy counterparts. Similar results were reported in a previous study in which the authors observed that MSCs derived from the skin of patients with psoriasis had a less evident effect on proliferation than activated PBMCs [15].

The skin is an organism's first line of defense. Previous reports have shown that the skin utilizes different mechanisms to prevent pathogens from entering; in the case of infection, the skin is responsible for mounting an adequate immune response that preserves and/or restores tissue homeostasis [40, 41]. Likely due to such protective mechanisms, we observed a decreased immunosuppression capacity in HD-MSCs compared with BM-MSCs, and we observed an even greater number of MSCs in the epidermis that did not show immunosuppressive capacity. Nevertheless, this discrete immunosuppressive activity is necessary to maintain healthy skin because, as was observed in our results, such function is lost in NLD-MSCs, which together with the increase in HLA-1 expression may induce development of psoriatic lesions. Psoriatic skin lesions are characterized by an exacerbated inflammatory process, which according to previous reports would favor the immunosuppressive activity of MSCs [8]. Our results show that LD-MSCs recover their immunosuppressive capacity to levels observed in HD-MSCs.

**Figure 7:**
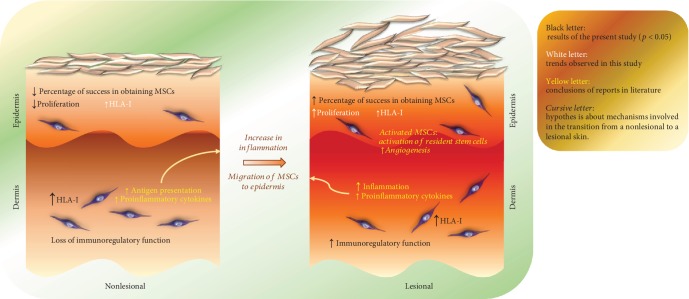
MSCs derived from the skin of patients with psoriasis show biological alterations. The nonlesional skin of patients with psoriasis already has a microenvironment that affects some characteristics of MSCs. The NLE-MSCs show alterations in their morphology and low proliferation. In addition, NLD-MSCs present a high HLA-I expression and do not have immunosuppression capacity against T lymphocytes; both of these aspects may be associated with an increase in antigen presentation, and therefore, such MSCs may be the initial stimuli for the overactivation of T cells and favor the development of psoriatic lesions. The exacerbated inflammation in lesional areas could favor the recruitment of MSCs and their activation. Several studies have shown that an inflammatory environment stimulates the secretion of trophic factors and immunosuppressive capacity by MSCs. Thus, the presence of MSCs in the lesional dermis and epidermis could contribute to the incomplete expansion and differentiation of epidermal stem cells; in addition, to their low immunosuppressive capacity, they would also favor the maintenance of these lesions.

In summary, our study presents several findings that may contribute to understanding of the pathophysiology of psoriasis (Figure 7). We demonstrate that NLE-MSCs show alterations in morphology and low proliferation capacity, which are two characteristics that have been associated with cellular senescence. In addition, NLD-MSCs present high HLA-I expression and do not have immunosuppression capacity against T lymphocytes; both of these aspects may be associated with an increase in antigen presentation, and therefore, such MSCs may be the initial stimulus for hyperactivation of T cells and favor the development of psoriatic lesions.

Psoriatic skin lesions are characterized by an exacerbated inflammatory reaction [1–3]. In this type of microenvironment, MSC recruitment could be induced [8, 42], thereby promoting an increase in the percentage of MSCs, as we observed in the lesional epidermis, and the MSCs may even show osteogenic differentiation potential. In contrast, in LD-MSCs, HLA-I expression is maintained at high levels and immunosuppressive capacity is recovered to the levels observed in healthy dermis but do not increase. Therefore, such capacity could be insufficient to resolve psoriatic lesions. IL-17 is currently known as the primary cytokine involved in development of psoriatic skin lesions [43, 44] and was recently shown to decrease the immunosuppressive capacity of MSCs [45]. Moreover the presence of proinflammatory cytokines has been shown to stimulate the secretion of trophic factors in MSCs that favor tissue remodeling [30]. Thus, the presence of MSCs in lesional dermis could contribute to incomplete expansion and differentiation of epidermal stem cells; in addition to their low immunosuppressive capacity, these MSCs might favor maintenance of psoriatic lesions (Figure 7).

## 5. Conclusion

To the best of our knowledge, this is the first study that analyzes the presence and biological characteristics of MSCs in dermis and epidermis from lesional and nonlesional skin. We observed a lower frequency of MSCs in the nonlesional epidermis, and the cells that developed in these cultures showed morphology alterations and low proliferation capacity. The differences that we found in the HLA-I expression levels in NLD-MSCs and LD-MSCs suggest that these cells are involved in pathogenesis of the disease and may be the initial stimulus for overactivation of T cells. This idea is supported by the high HLA-I expression observed in NLD-MSCs and their low immunoregulatory capacity against CD3^+^ T cells. Overall, our results indicate that in the dermis and epidermis of nonlesional areas, there is already a microenvironment that modifies the biological properties of MSCs and that may favor lesion development in patients with psoriasis.

## Figures and Tables

**Table 1:** The number of samples that were processed from bone marrow and the skin samples of healthy donors or patients with psoriasis, as well as the number of samples from which it was possible to establish a cell culture (percentage of success in obtaining MSCs).

Donor	Sample	Number of processed samples	Number of established MSC cultures	Percentage of success in obtaining MSCs
Healthy donors	Bone marrow (BM)	5	5	100
	Healthy dermis (HD)	5	5	100
	Healthy epidermis (HE)	5	5	100

Patients with psoriasis	Nonlesional dermis (NLD)	30	28	93
	Lesional dermis (LD)	20	19	95
	Nonlesional epidermis (NLE)	21	3	14
	Lesional epidermis (LE)	23	17	74

## Data Availability

The morphology, proliferation, immunophenotype, differentiation, and T cell proliferation data used to support the findings of this study are included within the article.
